# Supplementary material for: A comprehensive outline of antimicrobial resistance, antibiotic prescribing, and antimicrobial stewardship in South Africa: A scoping review protocol
Source: PLoS One. 2025 Jan 27;20(1):e0316718. doi: 10.1371/journal.pone.0316718 (PMC11771927; doi:10.1371/journal.pone.0316718)
Supplement: S1 Appendix — (DOCX) [file pone.0316718.s001.docx]

**Appendix 2: Search strategy developed for PubMed for scoping review (Search 12/08/2023)**

| **Search** | **Search term** | **Results** |
| --- | --- | --- |
| 1 | (“Antimicrobial Resistance” OR “Antibiotic Resistance” OR “Drug Resistance, Microbial” OR “Antimicrobial Stewardship” OR “Antibiotic Stewardship” OR “Resistance, Microbial” OR “Antibiotic Resistance Patterns” OR “Antimicrobial Resistance Surveillance” OR “AMR Control” OR “AMR in Healthcare” OR AMR knowledge” OR “AMR awareness” OR “Antimicrobial resistance awareness” OR “AMR education” OR “AMR training” OR “AMR understanding” OR “Antibiotic resistance awareness among healthcare workers’ OR ‘Resistance patterns” OR “Antimicrobial resistance pathogens” OR “Antibiotic resistance trends” OR “Pathogen resistance” OR “AMR pathogen prevalence” OR “Drug-resistant pathogens” OR “Infection control and AMR”) | 40074 |
| 2 | (“Surveillance” OR “AMR surveillance” OR “Antimicrobial resistance surveillance” OR “Antibiotic surveillance” OR “AMR monitoring” OR “Antimicrobial resistance control” OR “AMR management” OR “Antimicrobial resistance reporting” OR “AMR in healthcare facilities” OR “Resistance monitoring” OR “Surveillance systems for AMR”) | 10099 |
| 3 | (“Healthcare” OR “Healthcare workers” OR “Health professionals” OR “Healthcare settings” OR “Hospitals” OR “Primary Care” OR “Prescribers” OR “Medical staff” ) | 392 |
| 4 | Antibiotic prescribing” OR “Antimicrobial prescribing” OR “Antibiotic stewardship practices” OR “Antimicrobial stewardship” OR “Prescribing patterns” OR “Antibiotic guidelines” OR “Antibiotic prescribing behaviors” OR “Antimicrobial drug prescribing”) | 57 |
| 5 | (“South Africa” OR “South African” OR “South Africa healthcare”) | 51 |
|  | #1 AND #2 AND #3 AND #4 | 42 |
