# Supplementary material for: A comprehensive outline of antimicrobial resistance, antibiotic prescribing, and antimicrobial stewardship in South Africa: A scoping review protocol
Source: PLoS One. 2025 Jan 27;20(1):e0316718. doi: 10.1371/journal.pone.0316718 (PMC11771927; doi:10.1371/journal.pone.0316718)
Supplement: Attachment — Submitted filename: Highlighted corrected PONE-D-24-33778_reviewer.pdf [file pone.0316718.s005.pdf]

PLOS ONE

A Comprehensive Outline of Antimicrobial Resistance, Antibiotic Prescribing, and Antimicrobial Stewardship in South Africa: A Scoping Review Protocol

--Manuscript Draft--

**Manuscript Number:**	PONE-D-24-33778
**Article Type:**	Research Article
**Full Title:**	A Comprehensive Outline of Antimicrobial Resistance, Antibiotic Prescribing, and Antimicrobial Stewardship in South Africa: A Scoping Review Protocol
**Short Title:**	AMR, Antibiotic prescribing, Antimicrobial Stewardship in South Africa
**Corresponding Author:**	Suwayda Ahmed<br>University of the Western Cape<br>Cape Town, Western Cape SOUTH AFRICA
**Keywords:**	antimicrobial resistance, antimicrobial stewardship, antibiotic prescribing, scoping review, South Africa
**Abstract:**	Introduction The global prevalence of antimicrobial resistance transcends geographical and economic boundaries, affecting populations worldwide. Excessive and incorrect use of antibiotics encourages antimicrobial resistance which leads to complex treatment strategies for infectious diseases and possible failure of treatment. The incorrect and unnecessary prescribing of antibiotics places a burden on healthcare costs and thus, antimicrobial resistance is evident globally as a major public health concern. The impact is particularly pronounced in low to middle-income countries, where limited access to healthcare exacerbates the crisis. This scoping review aims to comprehensively map the evidence of antimicrobial resistance in healthcare settings, encompassing the exploration of antibiotic prescribing practices and the implementation of antimicrobial stewardship initiatives in South Africa. Methodology This protocol has been registered in the Open Science Framework (https://doi.org/10.17605/OSF.IO/PWMFB). This review will consider all types of study designs, conducted within South Africa. Studies that are published in English for the period 2019-2024, and that explore AMR evidence in healthcare in South Africa, including antibiotic prescribing trends and antimicrobial stewardship and surveillance initiatives will be included. Non-English publications, studies outside of South Africa, animal and environmental studies will be excluded. The criteria for the scoping review will be set by two reviewers. The PRISMA extension for Scoping Reviews tool will be used. Studies will be identified through an extensive search of peer-reviewed and grey literature databases. The results of the review will be tabulated and include a narrative synthesis of the findings.
**Order of Authors:**	Suwayda Ahmed
	Rukshana Ahmed
	Razia Zulfikar Adam
**Additional Information:**	
**Question**	**Response**
**Financial Disclosure**<br><br>Enter a financial disclosure statement that describes the sources of funding for the work included in this submission. Review the submission guidelines for detailed requirements. View published research articles from *PLOS ONE* for specific examples.	The author(s) received no specific funding for this work.

Powered by Editorial Manager® and ProduXion Manager® from Aries Systems Corporation

This statement is required for submission and **will appear in the published article** if the submission is accepted. Please make sure it is accurate.<br><br>**Funded studies**<br>Enter a statement with the following details:<br>• Initials of the authors who received each award<br>• Grant numbers awarded to each author<br>• The full name of each funder<br>• URL of each funder website<br>• Did the sponsors or funders play any role in the study design, data collection and analysis, decision to publish, or preparation of the manuscript?<br><br>Did you receive funding for this work?	
**Competing Interests**<br><br>Use the instructions below to enter a competing interest statement for this submission. On behalf of all authors, disclose any competing interests that could be perceived to bias this work—acknowledging all financial support and any other relevant financial or non-financial competing interests.<br><br>This statement is required for submission and **will appear in the published article** if the submission is accepted. Please make sure it is accurate and that any funding sources listed in your Funding Information later in the submission form are also declared in your Financial Disclosure statement.<br><br>View published research articles from *PLOS ONE* for specific examples.	The authors declare no conflict of interest

**NO authors have competing interests**<br><br>Enter: *The authors have declared that no competing interests exist.*<br><br>**Authors with competing interests**<br><br>Enter competing interest details beginning with this statement:<br><br>*I have read the journal's policy and the authors of this manuscript have the following competing interests: [insert competing interests here]*<br><br>* typeset	
**Ethics Statement**<br><br>Enter an ethics statement for this submission. This statement is required if the study involved:<br><br>• Human participants<br>• Human specimens or tissue<br>• Vertebrate animals or cephalopods<br>• Vertebrate embryos or tissues<br>• Field research<br><br>Write "N/A" if the submission does not require an ethics statement.<br><br>General guidance is provided below. Consult the submission guidelines for detailed instructions. **Make sure that all information entered here is included in the Methods section of the manuscript.**	Ethical approval for this project was obtained from the Biomedical Research Committee at the University of the Western Cape, (BMREC Reference Number: BM24/4/6).

Powered by Editorial Manager® and ProduXion Manager® from Aries Systems Corporation

**Format for specific study types**<br><br>**Human Subject Research (involving human participants and/or tissue)**<br>• Give the name of the institutional review board or ethics committee that approved the study<br>• Include the approval number and/or a statement indicating approval of this research<br>• Indicate the form of consent obtained (written/oral) or the reason that consent was not obtained (e.g. the data were analyzed anonymously)<br><br>**Animal Research (involving vertebrate animals, embryos or tissues)**<br>• Provide the name of the Institutional Animal Care and Use Committee (IACUC) or other relevant ethics board that reviewed the study protocol, and indicate whether they approved this research or granted a formal waiver of ethical approval<br>• Include an approval number if one was obtained<br>• If the study involved *non-human primates*, add *additional details* about animal welfare and steps taken to ameliorate suffering<br>• If anesthesia, euthanasia, or any kind of animal sacrifice is part of the study, include briefly which substances and/or methods were applied<br><br>**Field Research**<br><br>Include the following details if this study involves the collection of plant, animal, or other materials from a natural setting:<br>• Field permit number<br>• Name of the institution or relevant body that granted permission	
**Data Availability**<br><br>Authors are required to make all data underlying the findings described fully available, without restriction, and from the time of publication. PLOS allows rare exceptions to address legal and ethical concerns. See the PLOS Data Policy and FAQ for detailed information.	No - some restrictions will apply

Powered by Editorial Manager® and ProduXion Manager® from Aries Systems Corporation

A Data Availability Statement describing where the data can be found is required at submission. Your answers to this question constitute the Data Availability Statement and **will be published in the article**, if accepted.<br><br>**Important:** Stating 'data available on request from the author' is not sufficient. If your data are only available upon request, select 'No' for the first question and explain your exceptional situation in the text box.<br><br>Do the authors confirm that all data underlying the findings described in their manuscript are fully available without restriction?	
**Describe where the data may be found in full sentences. If you are copying our sample text, replace any instances of XXX with the appropriate details.**<br><br>• If the data are **held or will be held in a public repository**, include URLs, accession numbers or DOIs. If this information will only be available after acceptance, indicate this by ticking the box below. For example: *All XXX files are available from the XXX database (accession number(s) XXX, XXX.).*<br>• If the data are all contained **within the manuscript and/or Supporting Information files**, enter the following: *All relevant data are within the manuscript and its Supporting Information files.*<br>• If neither of these applies but you are able to provide **details of access elsewhere**, with or without limitations, please do so. For example:<br><br>*Data cannot be shared publicly because of [XXX]. Data are available from the XXX Institutional Data Access / Ethics Committee (contact via XXX) for researchers who meet the criteria for access to confidential data.*<br><br>*The data underlying the results presented in the study are available from (include the name of the third party*	Data generated or analysed in this study is included in this published article (and its supplementary information).

and contact information or URL).<br>• This text is appropriate if the data are owned by a third party and authors do not have permission to share the data.<br><br>* typeset	
Additional data availability information:	Tick here if the URLs/accession numbers/DOIs will be available only after acceptance of the manuscript for publication so that we can ensure their inclusion before publication.

Manuscript

Click here to access/download;Manuscript;Scoping Review Protocol Manuscript.docx

MANUSCRIPT TITLE:

A Comprehensive Outline of Antimicrobial Resistance, Antibiotic Prescribing, and Antimicrobial Stewardship in South Africa: A Scoping Review Protocol

AUTHORS DETAILS:

1. DR SUWAYDA AHMED
BChD, PDD, MSc
Senior Lecturer
Department of Prosthodontics
Faculty of Dentistry
University of the Western Cape
Cape Town, South Africa
Email address: suahmed@uwc.ac.za
Tel no.: (021)9373091
ORCID no: 0000-0001-8174-6928

2. DR RUKSHANA AHMED
BChD, PDD, MSc
Senior Lecturer
Department of Prosthodontics
Faculty of Dentistry
University of the Western Cape
Cape Town, South Africa
Email address: rahmed@uwc.ac.za
Tel no.: (021) 9373094
ORCID no: 0000-0002-0286-9047

3. PROF RAZIA ZULFIKAR ADAM
BChD, MSc, PhD
Associate Professor
Department of Prosthodontics

Faculty of Dentistry
University of the Western Cape
Cape Town, South Africa
Email address: rzadam@uwc.ac.za
Tel no.: (021) 9373003
ORCID no: 0000-0002-2645-9878

CORRESPONDING AUTHOR:

DR SUWAYDA AHMED

Department of Prosthodontics
Faculty of Dentistry
University of the Western Cape
Email address: suahmed@uwc.ac.za
Tel no.: (021)9373091
Cell no.: +27 825375827

Postal Address:
Private Bag X1
Tygerberg 7505
Cape Town
South Africa

A Comprehensive Outline of Antimicrobial Resistance, Antibiotic Prescribing, and Antimicrobial Stewardship in South Africa: A Scoping Review Protocol

ABSTRACT

Introduction

The global prevalence of antimicrobial resistance transcends geographical and economic boundaries, affecting populations worldwide. Excessive and incorrect use of antibiotics encourages antimicrobial resistance which leads to complex treatment strategies for infectious diseases and possible failure of treatment. The incorrect and unnecessary prescribing of antibiotics places a burden on healthcare costs and thus, antimicrobial resistance is evident globally as a major public health concern. The impact is particularly pronounced in low to middle-income countries, where limited access to healthcare exacerbates the crisis. This scoping review aims to comprehensively map the evidence of antimicrobial resistance in healthcare settings, encompassing the exploration of antibiotic prescribing practices and the implementation of antimicrobial stewardship initiatives in South Africa.

Methodology

This protocol has been registered in the Open Science Framework (https://doi.org/10.17605/OSF.IO/PWMFB). This review will consider all types of study designs, conducted within South Africa. Studies that are published in English for the period 2019-2024, and that explore AMR evidence in healthcare in South Africa, including antibiotic prescribing trends and antimicrobial stewardship and surveillance initiatives will be included. Non-English publications, studies outside of South Africa, animal and environmental studies will be excluded. The criteria for the scoping review will be set by two reviewers. The PRISMA extension for Scoping Reviews tool will be used. Studies will be

identified through an extensive search of peer-reviewed and grey literature databases. The results of the review will be tabulated and include a narrative synthesis of the findings.

INTRODUCTION

The World Health Organization (WHO) has highlighted antimicrobial resistance (AMR) as a life-threatening global health threat [1]. AMR is driven by various factors, of which the overuse of antibiotics is the most dominant [2]. Inappropriate prescribing and excessive use of antibiotics are a major driver of AMR worldwide [3-6]. In 2019, antimicrobial drug resistance infections contributed to 4.95 million deaths globally, with 1,27 million deaths attributable to bacterial AMR. Low to middle income countries (LMICs), especially in Sub-Saharan Africa are especially vulnerable to the threat of AMR [2]. Between 2000 and 2015, LMICs have seen a substantial increase in AMR rates due to the lack of access to clean water, sanitation, and hygiene [1]. Drivers of AMR in LMICs are influenced by political, economic, socio-cultural, and ecological factors that influence the profile of these countries [7].

Managing AMR in low- and middle-income countries, such as those in Africa prove challenging when compared to their high-income counterparts. Sub-Saharan African countries efforts to implement effective and workable AMR stewardship programs are often disordered by various factors; which include lack of human resources, decreased investment, decreased infrastructural and institutional capacities [8-9]. Lack of data around AMR complicates the management of AMR in Sub Saharan Africa [9]. Country specific data is not routinely assembled and is often not shared with national regulatory bodies. This places limitations on the ability of these regulatory bodies to effect national action [10-11].

South Africa, which is categorised as a middle-income country is not exempt of the threat of AMR, which manifests itself in increased mortality rates, extended hospitalization, escalated cost of healthcare and expenditure of healthcare resources [12-13].

Addressing the threat of AMR requires action targeted at the following aspects:

- Prevention of infection and the management of microbial transmission
- Improvement of antibiotic use via surveillance, as well as use of prescribing guidelines amongst prescribers
- Antimicrobial stewardship methods to stop the spread of resistant organisms [14].

In recognizing the threat of AMR, especially as it relates to LMICs, the National Department of Health in South Africa drafted the National AMR Strategy Framework [15]. This framework aligns with the WHO Global Action Plan as it underscores interdisciplinary efforts, antimicrobial stewardship and the elevation of infection prevention and control [16]. The aim of this national strategy framework is to provide guidance in order to limit the increase of antibacterial resistance and improve the standard of health amongst the general population. One of the strategic objectives of the strategy framework is to promote the accurate use of antibiotics in humans, and thus introduce antimicrobial stewardship at institutional levels. This would include AMS programmes at every health care facility, and district to regulate appropriate prescribing. In addition, reduction of antibiotic prescribing and workforce development also features amongst the objectives of the national strategy framework. The emphasis here is to facilitate the education of AMR through teaching and continuous training exercises [15].

The South African healthcare system is varied, and consists of a public health sector which is funded by the national government and a private health sector which is funded via medical insurance or private individuals. The majority of the South African population rely on the public healthcare sector to access healthcare [17-20]. The lack of resources, limited microbiological laboratory testing, delay in feedback of results and poor infrastructure are factors which hinder the accurate description of AMR rates in South Africa [9,21].

Healthcare facilities in South Africa are at varying phases of antimicrobial surveillance and stewardship. Studies confirmed that although healthcare facilities and prescribers adhered to antibiotic prescribing guidelines, inappropriate prescribing is still evident amongst South African prescribers [21-23].

Rationale for scoping review

South Africa, which is categorised as a middle-income country in Sub-Saharan Africa, faces significant challenges regarding the public health crisis of AMR. Antimicrobial stewardship practices vary across healthcare sectors despite the presence of guidelines, contributing to inappropriate prescribing behaviours. The National Department of Health has identified prevalent pathogens responsible for resistance patterns among South African populations, guiding local efforts in antimicrobial surveillance and stewardship [24]. Initial literature searches have confirmed existing reviews on antimicrobial stewardship and AMR across Africa [10,25,26]. This scoping review is aimed at taking an all-encompassing view at AMR rates and evidence, antibiotic prescribing trends among prescribers as well as surveillance measures and stewardship programmes in place in South Africa.

METHODS AND ANALYSIS

Review Question

What is the current status of AMR in South Africa; incorporating AMR awareness, antibiotic prescribing practices and antimicrobial stewardship?

Methodology

The reviewers (SA and RA) will follow the Preferred Reporting Items for Systematic Reviews and Meta-Analysis (PRISMA-SCR) approach for selection of articles on the current AMR situation, antibiotic knowledge and prescribing patterns and management of AMR [27].

The methodological framework by Arksey and O'Malley will be used to conduct this scoping review [28]. The following steps will be followed: identifying a clear research question; identifying related studies in literature; selection of articles; data extraction; summarise, synthesise and report on findings. The key research question leading this review is: What is known from the literature about AMR in healthcare in South Africa?

Search strategy

A comprehensive search strategy will be developed, using the following databases for peer-reviewed full text articles: PubMed, Scopus, Wiley, Directory of Open Access Journals and Science Direct and the WHO database. The Google search engine will be used to search for grey literature.

The following terms will be used in a combination of key Medical Subjects Heading (MeSH) terms and Boolean operators ("OR/AND"):

(Antibiotics OR antimicrobials) AND (Antimicrobial OR antibacterial resistance) AND Antimicrobial Stewardship AND Antibiotic Prescribing AND South Africa

Study selection

The two reviewers (SA and RA) will independently search through the literature, and assess the suitability of the studies from the databases as determined by the eligibility criteria. Study eligibility and data extraction forms will be used to guide the researchers to select the suitable documents and extract data independently. The two sets of literature will be compared. Any duplicate studies will be excluded manually. Studies where the study abstract does not relate to the focus question will be excluded. The two reviewers will independently screen titles and abstracts based on the predetermined inclusion and exclusion criteria. Thereafter, the two reviewers will independently assess the full text articles to determine the final eligibility. Any differing opinions will be resolved by consultation with the second reviewer, if required. A

third reviewer (RZA) will assist if consensus cannot be reached. Data extraction will be completed from the final full-text included documents obtained from the different databases.

Identification of relevant studies

The inclusion criteria for this scoping review will be based on the Population-Concept-Context (PCC) framework (Table 1).

Table 1. Arksey and O'Malley (2005) framework for eligibility of the research question

**Criteria**	**Determinants**
Population	Healthcare workers, undergraduate students and healthcare facilities
Concept	Evidence, knowledge and awareness of AMR<br>Antibiotic prescribing practices<br>Stewardship and surveillance of AMR
Context	Healthcare in South Africa

The population of interest in this scoping review will be healthcare workers and undergraduate healthcare students at healthcare facilities in South Africa. The concept will consist of evidence of AMR, awareness and knowledge of AMR among healthcare workers and students, antibiotic prescribing practices among healthcare workers, antimicrobial surveillance measures and antimicrobial stewardship interventions in health facilities.

Data extraction and charting

The data will be organized and reported on, according to themes as described in Table 2.

Table 2. Themes for data extraction and charting

**Key Themes**	**Sub-themes**
**The current antimicrobial resistance situation**	• AMR evidence<br>• Knowledge and awareness of AMR among HCWs and students<br>• Perception regarding rational prescribing of antimicrobials in relation to AMR
**Antibiotic knowledge, prescribing patterns and guidelines**	• Antibiotic prescribing patterns<br>• Knowledge of antimicrobial agents<br>• Compliance with antibiotic prescribing guidelines
**Management of AMR (stewardship and surveillance)**	• Stewardship policies<br>• Surveillance mechanisms<br>• Knowledge and awareness of management of AMR (stewardship and surveillance) among HCWs and students

The two reviewers (SA &RA) will extract data independently using a data extraction form (see appendix) which will collate the following information:

1. Title, author, year of publication
2. Study design
3. Study location
4. Themes/subthemes addressed
5. Key findings

Summarising and Reporting Results

The results will be outlined in a logical and descriptive summary that align with the objective/s of this review. The results will be mapped to the thematic framework as well as in descriptive form. The findings will be narratively summarised.

ABBREVIATIONS

AMR: antimicrobial resistance; Low to middle income countries: LMIC; Population-Concept-Context: PCC; Preferred Reporting Items for Systematic Reviews and Meta-Analysis: PRISMA-SCR; World Health Organization: WHO

ACKNOWLEDGMENTS

Not applicable

AUTHORS CONTRIBUTION

SA conceptualized the idea for the scoping review. SA and RZA developed the research questions. SA and RA developed the methodology, search strategy and data extraction table. SA, RA and RZA discussed the protocol design, edited and approved the final manuscript.

FUNDING

No external funding received.

AVAILIBILITY OF DATA

Data generated or analysed in this study is included in this published article (and its supplementary information).

DECLARATIONS

Ethics approval

Ethical approval for this project was obtained from the Biomedical Research Committee at the University of the Western Cape, (BMREC Reference Number: BM24/4/6).

Consent for publication

Not applicable.

Competing Interests

None declared.

Authors Details

[1]Department of Prosthodontics, Faculty of Dentistry, University of the Western Cape, Cape Town, South Africa; [2] Department of Prosthodontics, Faculty of Dentistry, University of the Western Cape, Cape Town, South Africa; [3]Department of Prosthodontics, Faculty of Dentistry, University of the Western Cape, Cape Town, South Africa.

REFERENCES

1. Walsh TR, Gales AC, Laxminarayan R, Dodd PC. Antimicrobial Resistance: Addressing a Global Threat to Humanity. *PLoS Med* 2023; 20(7):e1004264

2. Irfan M, Almotiri A, AlZeyadi ZA. Antimicrobial resistance and its drivers-A review. *Antibiotics* 2022;11(10):1362.
3. Tang KWK, Millar BC, Moore JE. Antimicrobial resistance (AMR). *Br. J. Biomed. Sci.* 2023; 80:11387.
4. Murray CJ, Ikuta KS, Sharara F, Swetschinski L, Aguilar GR, Gray A, Han C, Bisignano C, Rao P, Wool E, Johnson SC. Global burden of bacterial antimicrobial resistance in 2019: a systematic analysis. *Lancet* 2022; 399(10325):629-655.
5. Nisabwe L, Brice H, Umuhire MC, Gwira O, Harelimana JDD, Nzeyimana Z, Sebatunzi OR, Rusingiza EK, Hahirwa I, Muvunyi CM. Knowledge and attitudes towards antibiotic use and resistance among undergraduate healthcare students at University of Rwanda. *JOPPP* 2020; 13(1):1-8.
6. Sukumar S, Martin FE, Hughes TE, Adler CJ. 2020. Think before you prescribe: how dentistry contributes to antibiotic resistance. *Aust. Dent. J.* 2020; 65(1):21-29.
7. Sulis G, Sayood S, Gandra S. Antimicrobial resistance in low-and middle-income countries: current status and future directions. *Expert Rev. Anti. Infect.* 2022; 20(2):147-160.
8. Elton L, Thomason MJ, Tembo J, Velavan TP, Pallerla SR, Arruda LB, Vairo F, Montaldo C, Ntoumi F, Abdel Hamid MM, Haider N. Antimicrobial resistance preparedness in sub-Saharan African countries. *Antimicrob Resist& Infect Control* 2020; 9:1-11.
9. Chetty S, Reddy M, Ramsamy Y, Naidoo A, Essack S. Antimicrobial stewardship in South Africa: A scoping review of the published literature. *JAC-AMR* 2019; 1(3):60.
10. Okolie OJ, Igwe U, Ismail SU, Ighodalo UL, Adukwu EC. Systematic review of surveillance systems for AMR in Africa. *J. Antimicrob. Chemother* 2023; 78(1):31-51.

11. Porter GJ, Owens S, Breckons M. A systematic review of qualitative literature on antimicrobial stewardship in Sub-Saharan Africa. *GHJ* 2021; 6:1-11.

12. Sharma S, Chauhan A, Ranjan A, Mathkor DM, Haque S, Ramniwas S, Tuli HS, Jindal T, Yadav V. Emerging challenges in antimicrobial resistance: implications for pathogenic microorganisms, novel antibiotics, and their impact on sustainability. *Front microbiol* 2024; 15:1403168.

13. Chigome A, Ramdas N, Skosana P, Cook A, Schellack N, Campbell S, Lorenzetti G, Saleem Z, Godman B, Meyer JC. A narrative review of antibiotic prescribing practices in primary care settings in South Africa and potential ways forward to reduce antimicrobial resistance. *Antibiotics* 2023; 12(10):1540.

14. Aljeldah MM. Antimicrobial resistance and its spread is a global threat. *Antibiotics* 2022; 11(8):1082.

15. National Department of Health. *South African Antimicrobial Resistance National Strategy Framework; One Health Approach 2017-2024,* 2020 https://knowledgehub.health.gov.za/system/files/elibdownloads/2020-03/AMR%20National%20Action%20Plan%202018%20-%202024.pdf (Accessed March 2024)

16. World Health Organization. *Global action plan on antimicrobial resistance.* 2016 https://www.who.int/publications/i/item/9789241509763 (Accessed December 2023)

17. Meyer JC, Schellack N, Stokes J. Ongoing initiatives to improve the quality and efficiency of medicine use within the public healthcare system in South Africa; a preliminary study. *Front Pharmacol* 2017; 8:751.

18. Schellack N, Benjamin D, Brink A, Duse A, Faure K, Goff D, Mendelson M, Meyer J, Miot J, Perovic O, Pople T. A situational analysis of current antimicrobial governance, regulation, and utilization in South Africa. *IJID.* 2017; 64:100-106.

19. Schellack N, Meyer JC, Gous AG, Winters C. Part II. Health and economic context. *SAMJ.* 2011;101(sup-1):558-61.

20. Skosana PP, Schellack N, Godman B, Kurdi A, Bennie M, Kruger D, Meyer JC. A national, multicentre web-based point prevalence survey of antimicrobial use in community healthcare centres across South Africa and the implications. *Hosp. Pract.* 2022;50(4):306-317.

21. Chetty S, Reddy M, Ramsamy Y, Dlamini VC, Reddy-Naidoo R, Essack SY. Antimicrobial stewardship in public-sector hospitals in KwaZulu-Natal, South Africa. *Antibiotics* 2022;11(7):881.

22. Peters SM, Sheikh S, Werner JL, Morgan-Davies AM, Willems B. Antimicrobial stewardship in the Western Cape: A situational analysis of existing facility-level initiatives. *SAMJ.* 2021;111(5):421-425.

23. National Department of Health. *Surveillance for antimicrobial resistance and consumption of antimicrobials in South Africa* 2021https://knowledgehub.health.gov.za/system/files/elibdownloads/2023-04/AMR%2520and%2520AMC%2520report%2520for%25202021%2520in%2520South%2520African_June2022.pdf (Accessed March 2024)

24. Kamere N, Garwe ST, Akinwotu OO, Tuck C, Krockow EM, Yadav S, Olawale AG, Diyaolu AH, Munkombwe D, Muringu E, Muro EP. Scoping review of national antimicrobial stewardship activities in eight African countries and adaptable recommendations. *Antibiotics* 2022;11(9):1149.

25. Tadesse BT, Ashley EA, Ongarello S, Havumaki J, Wijegoonewardena M, González IJ. Antimicrobial resistance in Africa: a systematic review. *BMC Infect Dis.* 2017;17: 616.

26. Page MJ, McKenzie JE, Bossuyt PM, Boutron I, Hoffmann TC, Mulrow CD, Shamseer L, Tetzlaff JM, Akl EA, Brennan SE and Chou R. The PRISMA 2020 statement: an updated guideline for reporting systematic reviews. *BMJ* 2021;372.

27. Arksey H and O'Malley L. Scoping studies: towards a methodological framework. *Int. J. Soc. Res. Methodol* 2005;8(1):19-32.

Click here to access/download
Supporting Information
Appendix 1 PRISMA flow diagram.docx

Click here to access/download
Supporting Information
Appendix 2 PRISMA-ScR-Checklist.docx

Click here to access/download
Supporting Information
Appendix 3 Data extraction Table Template.xlsx

Click here to access/download
Supporting Information
Appendix 4 DATA COLLECTION ITEMS.docx

Click here to access/download
Supporting Information
PRISMA-P-SystRev-checklist PLOS ONE.docx
