# Supplementary material for: A comprehensive outline of antimicrobial resistance, antibiotic prescribing, and antimicrobial stewardship in South Africa: A scoping review protocol
Source: PLoS One. 2025 Jan 27;20(1):e0316718. doi: 10.1371/journal.pone.0316718 (PMC11771927; doi:10.1371/journal.pone.0316718)
Supplement: Attachment — Submitted filename: Plos One Letter for Rebuttal 13 Dec 2024.pdf [file pone.0316718.s007.pdf]

The Editor
PLOS ONE

13 December 2024

Dear Editor

Re: Resubmission of manuscript PONE-D-24-33778
A Comprehensive Outline of Antimicrobial Resistance, Antibiotic Prescribing, and Antimicrobial Stewardship in South Africa: A Scoping Review Protocol

Thank you for your feedback and recommendations regarding our submitted manuscript. The additional insights have been helpful in managing the requested revisions. I have tabulated all feedback and the revisions implemented in response to the peer reviewers' comments. All changes to the manuscript have been highlighted in yellow. Specific questions and information requested by reviewers have been addressed within the tabulated form as well.

FEEDBACK	Action/Revision
The inclusion and exclusion criteria (e.g., "studies published in English") could be better justified. While it is understandable that language barriers may limit accessibility, non-English studies could offer valuable insights, especially in multilingual settings like a Nation as South Africa. It would be beneficial to include a rationale for this exclusion.<br>Also, excluding animal and environmental studies might be overly restrictive. Many environmental and veterinary studies contribute to understanding AMR dynamics in healthcare settings, especially in LMICs. The authors should provide a more robust argument for excluding these studies or consider including them for a more holistic perspective.	Acknowledged.<br>Although South Africa has 11 eleven official languages, the language of the academic discourse and academic instruction in South Africa is English. English is the primary medium of communication in universities, research publications and academic conferences; while the other official languages are not commonly used in these contexts. For the purposes of this study, which focuses on academic literature and research in South Africa, only studies published in English will be considered.
Also, excluding animal and environmental studies might be overly restrictive. Many environmental and veterinary studies contribute to understanding AMR dynamics in healthcare settings, especially in LMICs. The authors should provide a more robust argument for excluding these studies or consider including them for a more holistic perspective.	Acknowledged.<br>The scoping review is focussed on healthcare settings in South Africa, to provide a comprehensive overview of the landscape of AMR within the South African healthcare context, highlighting existing knowledge, challenges, and gaps in the healthcare sector. The scoping review aims to serve as a foundation for a larger doctoral

	research project by establishing the context in which AMR is experienced within healthcare in South Africa. The purpose would thus be to gain a greater understanding of AMR in healthcare settings in South Africa, looking at the link to broader issues like incorrect antibiotic prescribing, infection control practices, public health strategies, and local resistance patterns. By limiting the scope to AMR in healthcare, this scoping review may provide a focused and relevant setting for understanding AMR in South Africa.
Abstract<br>Line 75: It is important to define abbreviations before they are used. Please define AMR before using it.<br>Line 79: Define PRISMA before the use of the abbreviation.<br>Introduction<br>Line 117: Define AMS before using the abbreviation.	Acknowledged.<br>This has been corrected<br>Page 3, line 75<br>Page 3-4, line 79-80<br>Page 5, line 118
There are research/review questions in two sections of the manuscript. What is the difference between the review question and the research question? Please clarify the difference between these two types of questions for the benefit of the reader.	Acknowledged.<br>The research question is broader and provides a guiding inquiry for the scoping review, providing direction and context for the study.<br>The review questions encourage a more specific inquiry into particular aspects of the topic to focus the scope of the review and answer the research question.
What are the author's thoughts about including all the questions in one section? Research questions typically tend to be at the end of the introduction or background section. A recommendation is to include the review/research questions for the scoping review at the end of the introduction section.<br>****The rationale for scoping review section could stay in position as the final part of the introduction section, following which all the questions from lines 131-132 and 146-147 could be presented**.	Thank you for recommendation<br>We have relocated the research and review questions to the methodology section, ensuring that all questions are consolidated in one section ****** of the manuscript making it easier for the reader to recognize.<br>Page 6, line 146-150
Line 156-164: Search strategy. Previous feedback about the search strategy has not been addressed. There is a need for more	Acknowledged<br>The following inclusion will be used: Full-text peer-reviewed journal articles including

information in the inclusion and exclusion criteria. Would the year of publication be part of the search criteria?	study designs (quantitative, qualitative, observational, mixed methods studies) which are published in English, grey literature, and human studies for the period of 2019-2024<br>The exclusion criteria are as follows: Exclusion Criteria: Non-English articles, systematic reviews, narrative reviews, studies outside of South Africa, animal and environmental studies.<br>Narrative and systematic reviews will be excluded as they characteristically synthesize existing primary research and this may result in duplication of findings.<br>Page 7-8, line 168-172
What specific types of study designs would be included in the scoping review? How would the reviewers manage including data from different types of studies? For example, how would data from quantitative and qualitative studies; as well as data from cross-sectional studies and systematic reviews/meta-analysis be managed and analyzed for reporting?	Acknowledged<br>Explanation has been added to manuscript. Please see inclusion criteria on page 7-8 and also see pages 9-10, lines 200-210
Line 163-164: Would "healthcare workers" referenced in Line 191, rather than "healthcare" currently included as a Boolean term be used as a Boolean term for the literature search?	Acknowledged.<br>Healthcare would encompass a wider range of studies which could also relate to healthcare systems, policies and practices, in order to look at AMR and related factors that influence it.
A recommendation for smoother flow in reading information in the paper is to include the inclusion and exclusion criteria as well as the identification of relevant studies in the search strategy section. The study selection section can come after this section and lead to the data extraction and charting section.	Thank you for recommendation. The inclusion and exclusion criteria have been moved to the search strategy section; followed by study selection, data extraction and charting section.
Previous feedback about the literature screening, data extraction and charting have not been addressed. What tool, application or platform would be used for screening data extraction of the selected papers?	Acknowledged and completed<br>Additional detail has been added to the manuscript. The reviewers will be screening literature, data extraction and charting manually.<br>Page 11, line 215-222
Data extraction and charting<br>Lines 200-204: Information about key elements in the data extraction form would	Acknowledged<br>This paragraph has been rewritten. Please see page 11, line 215-222

look better in a paragraph format, rather than in the numbered format.	

I trust that we have adequately addressed all required edits and comments. I hope that you will view our revised manuscript favorably.

Kind regards

Dr Suwayda Ahmed
Department of Prosthodontics
Faculty of Dentistry
Tel: (021) 9373091
suahmed@uwc.ac.za
