# Supplementary material for: A comprehensive outline of antimicrobial resistance, antibiotic prescribing, and antimicrobial stewardship in South Africa: A scoping review protocol
Source: PLoS One. 2025 Jan 27;20(1):e0316718. doi: 10.1371/journal.pone.0316718 (PMC11771927; doi:10.1371/journal.pone.0316718)
Supplement: Attachment — Submitted filename: Reviewers comment.docx [file pone.0316718.s004.docx]

1. **Resaerch article type:**

I believe this should be a protocol instead of research article, as I understand that the main article is still currently on and not submitted for publication yet, so you might want to change it from the online submission form or kindly clarify.

2. **Keywords:**

Make sure each keyword begins with an uppercase letter, e.g., "Pain Management," "Animal Health," "Sustainable Materials," etc.

3. **Line-by-Line Corrections:**
a. **Lines 1-42** (Author and Affiliation Arrangement)

**Follow the PLOS ONE format:** List author names with the first name followed by the last name (e.g., "John Smith"). Each author should have a superscript number linking to their respective affiliations.

**Affiliation Format:** Include the department, institution, city, state, and country for each author. For example:

Department of Veterinary XXX, XXX University, Tuscaloosa, Alabam, USA.

Department of Animal Health, XYZ University, City, State, Country.

**Corresponding Author:** Mark with an asterisk and provide the email address after affiliations. For example:

*Corresponding Author: Dr. BA [(email@example.com)](mailto:(email@example.com))

b. **Lines 53-54 (Title, Abstract, and Headings Formatting)**

**Title:** Ensure it is in Title Case with only the first letter of each significant word capitalized, e.g., "Optimization of Low-Carbon Cementitious Materials for Pain Management in Veterinary Medicine."

**Abstract and Headings:** Format these according to PLOS ONE standards. Use bold for headings and ensure they are clear and succinct, e.g., Abstract, Introduction, Methods, Results, Discussion. Let there be clear difference between an heading and sub heading

c. **Line 84 (Numerical Formatting)**

Correct numerical representation, for example, “1.27 million” instead of “1,27 million” to align with English standards.

d. **Lines 102-105 (Collapsing Bulleted Sentences)**

Combine the bullet points into a coherent sentence:

"This study identifies several key factors including optimized design, improved material efficiency, and the integration of low-carbon cementitious materials, which together contribute to sustainable development in veterinary medical infrastructure."

e. **Line 129 (Methods and Analysis Subheading)**

Move any content under the "Introduction" that is methods-related to the "Methods and Analysis" section, ensuring a logical flow and proper context within the Methods section.

f. **Line 141 (Subheading Correction)**

Correct the subheading to "Review Questions" to ensure consistency and proper English usage.

g. **Lines 173-176 and 187-191 (Dead Space)**

Remove extra spaces between sections to maintain continuity and readability. Ensure the text flows seamlessly from one paragraph to the next.

**General Formatting Tips:**

1. **Paragraph Structure:** Ensure that each paragraph starts with a clear topic sentence and logically progresses to the conclusion.
2. **Grammar and Language:** Use active voice, concise language, and ensure subject-verb agreement throughout.
3. **References:** Follow PLOS ONE reference style, with in-text citations in numerical order as they appear, and full references formatted as per the journal's guidelines.

**All the best!!!**
