# Supplementary material for: A comprehensive outline of antimicrobial resistance, antibiotic prescribing, and antimicrobial stewardship in South Africa: A scoping review protocol
Source: PLoS One. 2025 Jan 27;20(1):e0316718. doi: 10.1371/journal.pone.0316718 (PMC11771927; doi:10.1371/journal.pone.0316718)
Supplement: Attachment — Submitted filename: R1 Reviewers comment.docx [file pone.0316718.s006.docx]

**Review of the Scoping Review Protocol on Antimicrobial Resistance, Antibiotic Prescribing, and Antimicrobial Stewardship in South Africa**

This protocol outlines a scoping review aiming to map the evidence on antimicrobial resistance (AMR), antibiotic prescribing practices, and antimicrobial stewardship (AMS) initiatives within South Africa. It aims to address the current state of AMR awareness, antibiotic prescribing patterns, and the implementation of AMS programs in healthcare settings. While the protocol offers a comprehensive approach, several areas could also benefit from further clarification, improvement, and refinement to strengthen the review's overall quality.

Although, the review addresses a highly pertinent global health issue, especially in low- and middle-income countries (LMICs), like South Africa and the aims and objectives of the review are clearly articulated, with a focus on key aspects of AMR in South Africa, including prescribing practices and stewardship efforts however, the inclusion and exclusion criteria (e.g., “studies published in English”) could be better justified. While it is understandable that language barriers may limit accessibility, non-English studies could offer valuable insights, especially in multilingual settings like a Nation as South Africa. It would be beneficial to include a rationale for this exclusion.

Also, excluding animal and environmental studies might be overly restrictive. Many environmental and veterinary studies contribute to understanding AMR dynamics in healthcare settings, especially in LMICs. The authors should provide a more robust argument for excluding these studies or consider including them for a more holistic perspective.
